# Reactivity and kinetics of 1,3-butadiene under ultraviolet irradiation at 254 nm

**DOI:** 10.1186/s13065-022-00800-6

**Published:** 2022-02-18

**Authors:** Min Liang, Chang Yu, Suyi Dai, Haijun Cheng, Weiguang Li, Fang Lai, Li Ma, Xiongmin Liu

**Affiliations:** School of Chemistry and Chemical Engineering, Guangxi University, Nanning, 530004 China

**Keywords:** 1,3-butadiene, Photolysis, Reaction kinetics, Ultraviolet irradiation

## Abstract

The reaction process of gaseous 1,3-butadiene following ultraviolet irradiation at the temperature range from 298 to 323 K under nitrogen atmosphere was monitored by UV–vis spectrophotometry. A gaseous mini-reactor was used as a reaction vessel and could be directly monitored in a UV–vis spectrophotometer. We investigated the reactivity and kinetics of 1,3-butadiene under non-UV and UV irradiation to evaluate its photochemical stability. A second-order kinetic model with 50.48 kJ·mol^–1^ activation energy fitted the reaction data for non-UV irradiation, whereas a first-order kinetic model was appropriate in the case of UV irradiation with activation energies of 19.92–43.65 kJ mol^–1^. This indicates that ultraviolet light could accelerate the photolysis reaction rate of 1,3-butadiene. In addition, the reaction products were determined using gas chromatography-mass spectrometry (GC–MS), and the reaction pathways were identified. The photolysis of 1,3-butadiene gave rise to various volatile products by cleavage and rearrangement of single C–C bonds. The differences between dimerization and dissociation of 1,3-butadiene under ultraviolet irradiation were elucidated by combining experimental and theoretical methods. The present findings provide fundamental insight into the photochemistry of 1,3-butadiene compounds.

**Supplementary Information:**

The online version contains supplementary material available at 10.1186/s13065-022-00800-6.

## Introduction

As an important organic precursor, 1,3-butadiene is widely used in the production of polybutadiene and other copolymers, such as *cis*-polybutadiene rubber [1, 2], neoprene, and styrene-butadiene polymers [3–5]. Owing to conjugation effects [6], 1,3-butadiene is prone to polymerize to form polymers and polyperoxides upon contact with light, heat, and oxygen in air [7], resulting in reduced performance and limiting its applications. Polyperoxides were reported to be impact-sensitive and thermally unstable, and slow deposition over some time can lead to highly hazardous conditions in 1,3-butadiene plants [8]. Many serious explosion accidents have occurred during the production of 1,3-butadiene [9–12]. Hendry et al. conducted impact experiments to determine the sensitivity of 1,3-butadiene polyperoxides using the standard drop weight method [13]. The results showed that a low-energy shock could cause rapid combustion, while a high-energy shock would produce a thermal explosion. Various types of calorimeters were used to study the thermal polymerization of 1,3-butadiene. The thermal characteristics and hazards associated with 1,3-butadiene were studied using an accelerated calorimeter (ARC) [14]. The thermal dimerization and polymerization reactions of 1,3-butadiene in the presence and absence of oxygen were evaluated to assess its thermal reactivity and runaway behavior using theoretical computational models and thermal analysis techniques [15]. An automated pressure-tracking adiabatic calorimeter (APTAC™) was used for measuring the overall thermodynamic and kinetic parameters.

In addition to thermal polymerization, the UV-based photopolymerization processes of 1,3-butadiene have also been extensively studied in the past decades. Earlier reports [16–19] showed that irradiation of solutions containing conjugated dienes and various photosensitizers led to the formation of dimers of the dienes. The photochemistry of 1,3-butadiene in solution yielded none of the main volatile products that were observed in the vapor phase but produced only cyclobutene, bicyclo[1.1.0]butane, dimers, and a polymer [20]. In addition to the photopolymerization products, some decomposition products, such as ethylene and acetylene were also detected the above study. However, photopolymerization and photolysis reactions involve two different mechanisms. The origin of the coexistence of these two competing mechanisms under ultraviolet light irradiation is worthy of further investigation by a combination theoretical and experimental methods. However, the specific requirements and testing procedures make the experimental analysis tedious and expensive. A more practical and simpler experimental analysis method should be employed, such as UV–vis spectrophotometry. On the other hand, the importance of the reaction rate in chemical production is self-evident. Understanding the effects of various factors on the reaction rate facilitates the selection of the conditions required to make the chemical reaction proceed at the desired rate. This highlights the crucial importance of investigating the kinetics of the photochemical reactions of gaseous 1,3-butadiene.

In this work, the reactions of gaseous 1,3-butadiene under UV irradiation in nitrogen atmosphere at low temperature were carried out in a gaseous ultraviolet mini-reactor. A UV–vis spectrophotometer was employed to monitor the reaction process. In order to clarify the influence of 254 nm UV light on the 1,3-butadiene reaction rate constants during the photochemical reaction, the reaction rate constants and activation energy obtained in absence of irradiation were used as reference. To understand the reactivity of 1,3-butadiene under ultraviolet irradiation, we studied the relationship between UV intensities and reaction rate constants. In addition, the reaction products were analyzed by gas chromatography–mass spectrometry (GC–MS), and the possible mechanisms of the photochemical reaction of 1,3-butadiene were systematically examined. The results were compared with the reaction mechanisms reported in the previous studies. The differences between the photolysis and photodimerization pathways for 1,3-butadiene under 254 nm irradiation are also discussed in detail.

## Material and methods

### Materials

1,3-Butadiene (mass purity > 99.90%, molecular weight 54.09 g mol^–1^) was obtained from Guangdong Walter Gas Co., Ltd., China. N_2_ gas (mass purity  > 99.99%) was obtained from Guangxi Guoxin Gas Research Co., Ltd., China.

### Photochemistry of 1,3-butadiene

**Fig. 1:**
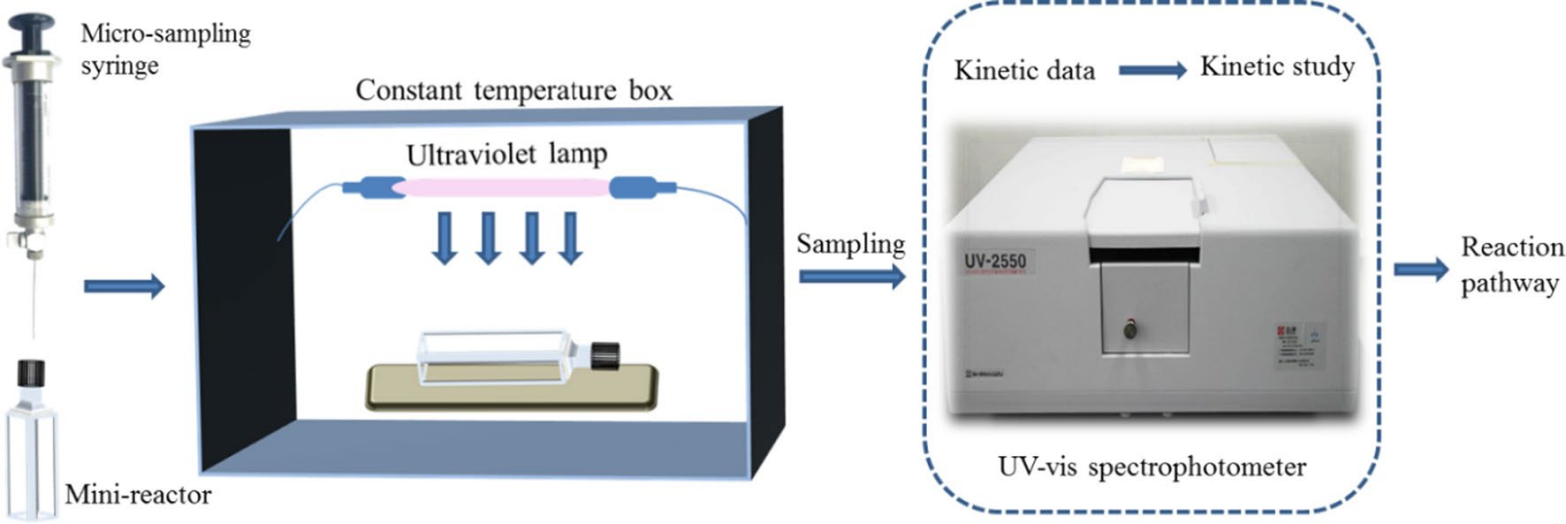
Schematic diagram of the research

A sealed threaded quartz colorimetric dish (volume 3.5 mL, Yixing Spectral Analysis Optical Components Co., Ltd., China) was used both as a gaseous ultraviolet mini-reactor and sample cell, which could be analyzed directly to avoid the errors associated with intermediate extraction. Before each experimental measurement, the reactor was first evacuated and simultaneously passed through fresh nitrogen for not less than 5 min to flush out all oxygen. The required trace amount of gaseous 1,3-butadiene was injected into the reactor using a gas micro-sampling syringe. Homogeneous mixing of the reactants in the reactor was carried out before starting the experiment. For quantitative analysis of 1,3-butadiene, the reactor was placed into a UV–vis spectrophotometer (UV-2550, Shimadzu Instruments Co., Ltd., Japan) at preselected time intervals. Then, the reactor was transferred into a constant-temperature box to maintain the temperature at the desired value (other than room temperature). A schematic diagram of the experiment is shown in Fig. 1. The photochemical reaction was performed at different temperatures (298, 303, 308, 313, 318, 323 K) for 5 h. The light source was a low-pressure UV lamp (Philips, TUV G6T5, 6 W) with maximum emission at 254 nm, which was placed on the top of the constant-temperature box. The light intensity of the UV lamp was between 25 and 500 μW cm^−2^, as measured by a UV-C ultraviolet radiometer (Shanghai Baoshan Gucun Optic Instrument Factory, Shanghai, China) in air. The intensity of the ultraviolet radiation was varied by adjusting the distance between the lamp and the reactor. Dynamic data processing was carried out using the iterative method (Additional file 1).

### Qualitative analysis of reaction products

The main products of the thermal and photochemical reactions of 1,3-butadiene were qualitatively analyzed by GC–MS (GC/MS-QP2010 Ultra, SHIMADZU, Japan) using an Agilent J&W advanced capillary column (30 m × 0.25 mm × 5.00 μm) and an electron impact ionization detector (EID, 70 eV). The analytical procedures were as follows: the temperature was maintained at 333 K for 1 min, then increased to 373 K at a rate of 3 K min^−1^, and kept for 3 min. The carrier gas was ultrahigh pure helium at a constant flow rate of 4.0 mL min^−1^. The injection temperature and volume were maintained at 373 K and 1.0 mL, respectively; the split ratio was 30:1, while the interface and ion source temperatures were set at 473 and 493 K, respectively. A quadrupole mass filter was used with a *m*/*z* range of 18–300 in full-scan mode. The detected peaks were identified based on the National Institute of Standards and Technology 2011 library of mass spectra. Qualitative analysis of the products was based on the cracking patterns and retention times observed in the mass spectrometry and gas chromatography analyses, respectively.

## Results and discussion

### Quantitative analysis of 1,3-butadiene by UV–vis spectrophotometry

**Fig. 2:**
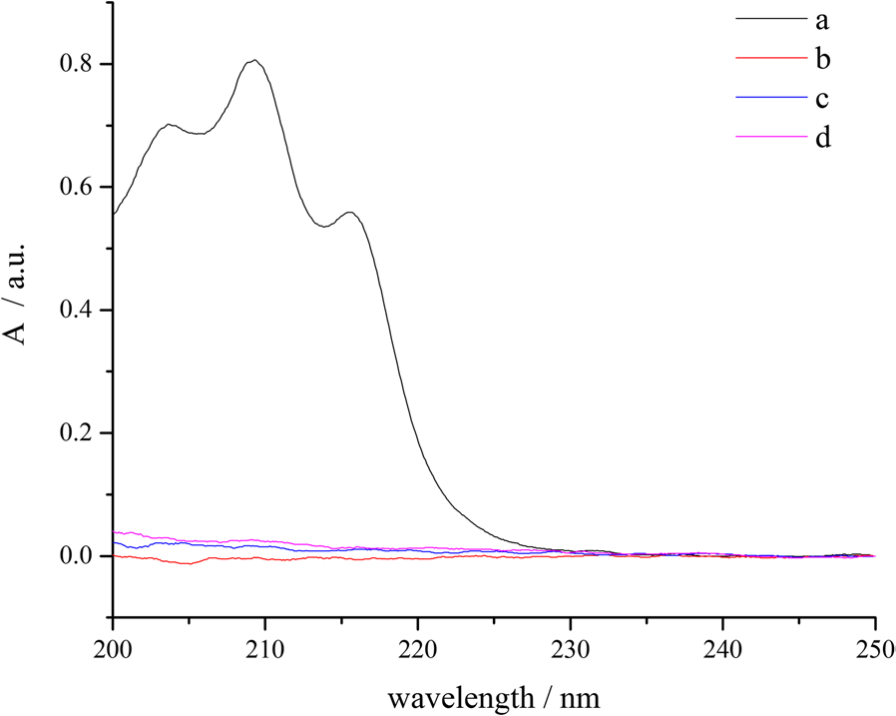
UV spectra of 1,3-butadiene reaction process. **a** 1,3-butadiene; **b** thermal products of 1,3-butadiene; **c** photoreaction products of 1,3-butadiene; **d** blank reactor

UV–vis spectrophotometry is widely employed for the quantitative determination of the concentration of liquids [21, 22]; however, it has rarely been applied to the analysis of other states of matter, such as gas. Therefore, in this study we aimed to fill this gap by using UV–vis spectrophotometry for the quantitative analysis of gaseous 1,3-butadiene. The experimental results are shown in Fig. 2. 1,3-Butadiene showed an obvious absorption peak with a maximum at 209 nm, due to the ultraviolet absorption of conjugated double bonds. Figure 2d shows that the reactor cell did not affect the process under experimental conditions. Therefore, quantitative analysis was performed spectrophotometrically by monitoring the decay of the strong absorbance peak of 1,3-butadiene at 209 nm. Figure 2b, c suggest that the absorption of the thermal and photoreaction products of 1,3-butadiene was negligible, because they showed almost no absorbance within the same wavelength range. The working equation for the quantitative analysis of 1,3-butadiene, as determined by the external standard method, was *y* = 0.4672 × 10^4^*x* + 0.1026 (*R*^2^ = 0.9996), where *y* and *x* represent the absorbance and amount (mol) of 1,3-butadiene, respectively. Then, the molar amount of 1,3-butadiene was calculated from the working curve equation. This approach provides a new route for the quantitative analysis of other gases as well.

### Thermal reaction kinetics of 1,3-butadiene

In order to clarify the influence of ultraviolet light on the reaction constants during the photochemical process, we first analysed the reaction in absence of ultraviolet illumination, that is, the thermal reaction. A detailed kinetic analysis was performed to assess the effect of the temperature on the rate of the thermal reaction of 1,3-butadiene. To simplify the kinetic equations and the calculation of the related parameters, gaseous 1,3-butadiene was considered to be uniformly distributed in the reactor.

The thermal reaction of 1,3-butadiene was assumed to proceed as follows:

$$\alpha \;{\text{CH}}_{2} = {\text{CH}}{-}{\text{CH}} = {\text{CH}}_{2} \;\underrightarrow {k_{1} }\;{\text{products}}$$

The reaction rate (*r*_1_) of 1,3-butadiene could be expressed as

1 $$r_{1} = - \frac{{dn_{BD} }}{dt} = k_{1} n_{BD}^{\alpha }$$

where *k*_1_ represents the kinetic rate constant of the thermal reaction, *t* is the reaction time (h), ${n}_{BD}$ is the amount of 1,3-butadiene (mol) at time *t*, and *α* denotes the thermal reaction order. Based on Eq. (1), the calculated values were fitted to different models via a trial-and-error method: i.e., a is first-order [Eq. (2)] or second-order [Eq. (3)]. We compared the correlation coefficient R^2^ to confirm the reaction order:

2 $$\ln n_{BD} = - k_{1} t + C$$

3 $$\frac{1}{{n_{BD} }} = - k_{1} t + C$$

where *C* is a constant.

**Fig. 3:**
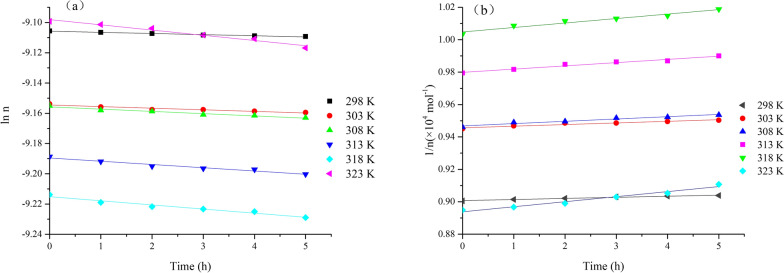
Correlation plots for the 1,3-butadiene thermal reaction, **a** first-order model; **b** second-order model

The experimental ln *n* vs. *t* or 1/*n* vs. *t* plots show a linear relationship, corresponding to the correct rate equation, and the rate constant was thus obtained from the slope of the regression line. The results are shown in Fig. 3 and the kinetic parameters are displayed in Table 1. It is indicating that the second-order model was more suitable for describing the 1,3-butadiene thermal reaction than the first-order model, suggesting that the thermal reaction of 1,3-butadiene is more like a second-order reaction. This is consistent with previous research [23]. It was suggested that the dimerization process followed a second-order kinetics in the temperature range from 298 to 323 K.

**Table 1:** Kinetic parameters for the 1,3-butadiene thermal reaction

T/K	First order model			Second order model		
	*k*_1_ × 10^–3^/h^−1^	Kinetic equation	R^2^	*k*_1_/(mol h)^−1^	Kinetic equation	R^2^
298	0.7504	ln n = − 0.7504 × 10^−3^t − 9.106	0.9812	6.771	1/n = 6.771 t + 9007	0.9813
303	1.055	ln n = − 1.055 × 10^−3^t − 9.154	0.9186	9.999	1/n = 9.999 t + 9457	0.9190
308	1.493	ln n = − 1.493 × 10^−3^t − 9.156	0.9565	14.19	1/n = 14.19 t + 9469	0.9569
313	2.149	ln n = − 2.149 × 10^−3^t − 9.190	0.9599	19.98	1/n = 19.98 t + 9799	0.9689
318	2.727	ln n = − 2.727 × 10^−3^t − 9.215	0.9596	27.58	1/n = 27.58 t + 10,048	0.9602
323	3.449	ln n = − 3.449 × 10^−3^t − 9.098	0.9716	31.12	1/n = 31.12 t + 8938	0.9708

The activation energies (*E*_a_) could be estimated using the Arrhenius equation:

4 $$k = A_{0} \exp ( - E_{a} /RT)$$

where *k* is the rate constant, *R* is the gas constant [8.314 J·(mol K)^−1^], *A*_0_ is the frequency factor, and *T* is the absolute temperature. Taking the natural logarithm of both sides of Eq. (4), we obtain

5 $$\ln k = - \frac{{E_{a} }}{RT} + \ln A_{0}$$

**Fig. 4:**
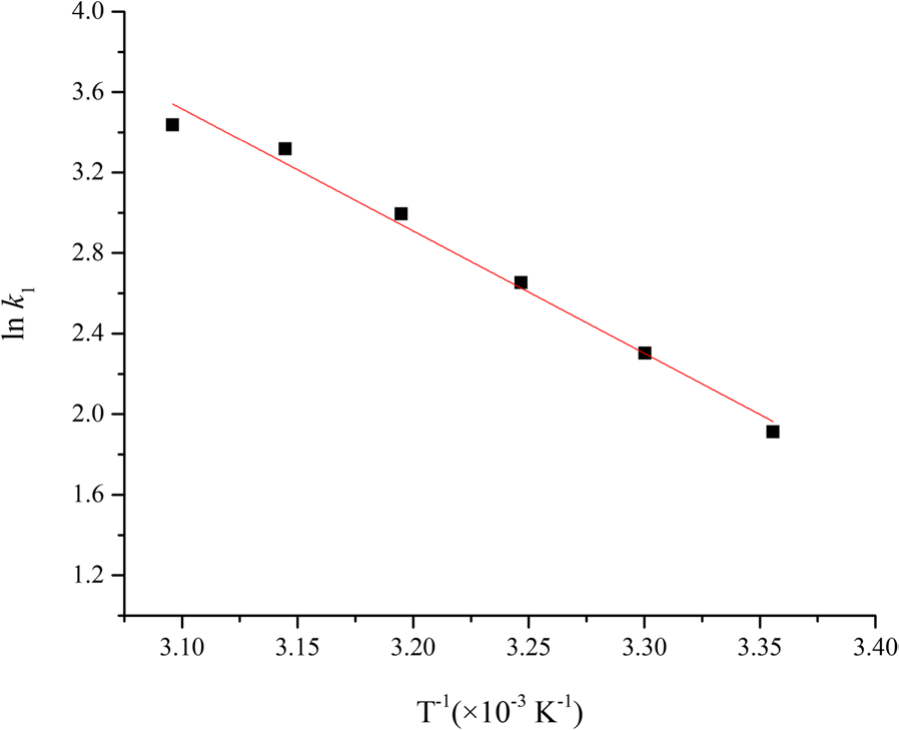
The plot of ln *k*_1_ vs. *T*^−1^

Plots of ln *k* vs. *T*^−1^ consisted of a straight line (Fig. 4), whose slope was used to calculate the activation energy of the thermal reaction of 1,3-butadiene, which was estimated to be 50.48 kJ mol^−1^.

### Photolysis kinetics of 1,3-butadiene under ultraviolet irradiation

The photochemical reaction of 1,3-butadiene can be represented as follows:

$$\beta \;{\text{CH}}_{2} = {\text{CH}}{-}{\text{CH}} = {\text{CH}}_{2} \underrightarrow {hv,k_{2} }\;{\text{products}}$$

The general photolysis reaction rate expression (*r*_2_) could be written as

6 $$r_{2} = - \frac{{dn_{BD} }}{dt} = k_{2} n_{BD}^{{\upbeta }}$$

where *k*_2_ and *β* denote the rate constant and order of the photolysis reaction.

**Fig. 5:**
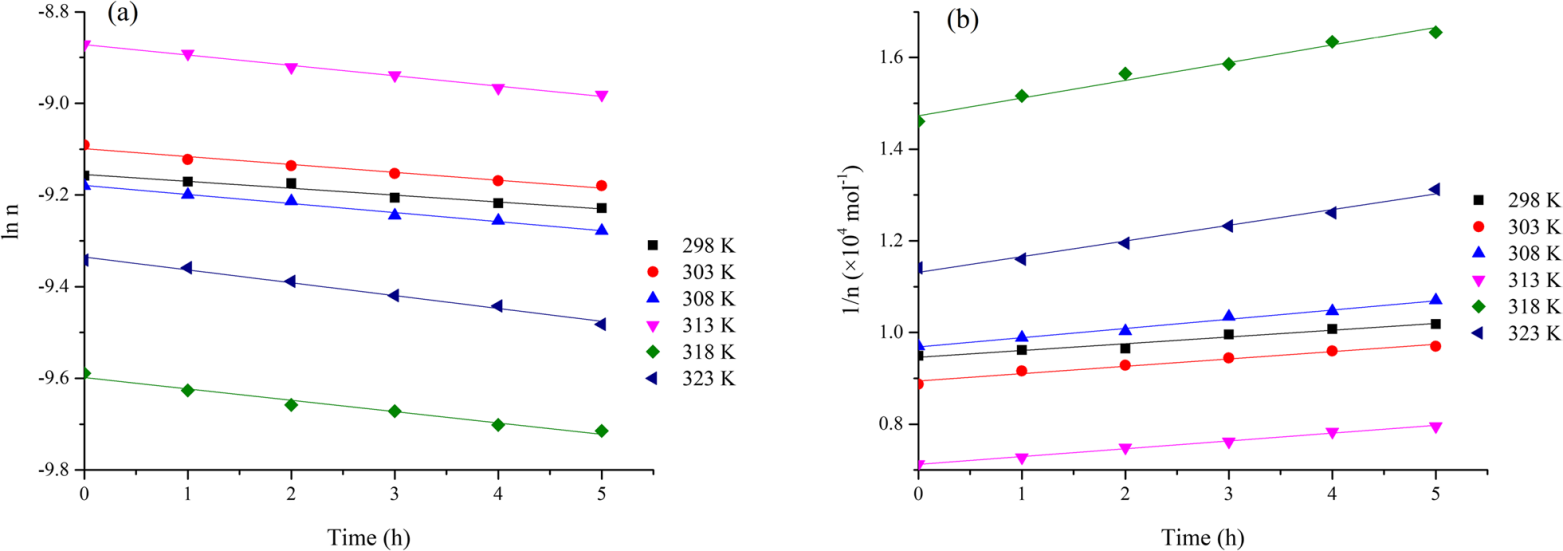
Curves of 1,3-butadiene photolysis, **a** first-order model; **b** second-order model

Similar to the thermal reaction kinetics of 1,3-butadiene, two kinds of kinetics model were plot in Fig. 5. The kinetic parameters of the 1,3-butadiene photoreaction are shown in Table 2. It can be inferred from this data that the photolysis reaction follows a first-order kinetics. Rauchenwald et al. reported a new method of destroying waste anesthetic gases by using gas-phase photochemistry and the photochemistry exhaust gas destruction system exhibits a constant first-order removal rate [24]. Hu et al. investigated the VUV/UV photodegradation of three iodinated disinfection byproducts followed pseudo-first-order kinetics [25]. The 1,3-butadiene photolysis under 254 nm UV is well fitted by first-order kinetics. The activation energy of the photolysis process was calculated to be 19.92 kJ mol^−1^.

**Table 2:** Kinetic parameters of 1,3-butadiene photolysis

T/K	First order model			Second order model		
	*k*_2_ × 10^–2^/h^−1^	Kinetic equation	R^2^	*k*_2_/(mol h)^−1^	Kinetic equation	R^2^
298	1.102	ln n = − 1.102 × 10^–2^ t − 7.394	0.9599	147.6	1/n = 147.6 t + 9460	0.9596
303	1.282	ln n = − 1.282 × 10^–2^ t − 7.352	0.9714	160.2	1/n = 160.2 t + 8943	0.9706
308	1.436	ln n = − 1.436 × 10^–2^ t − 7.412	0.9902	201.2	1/n = 201.2 t + 9686	0.9898
313	1.574	ln n = − 1.574 × 10^–2^ t − 7.703	0.9729	170.4	1/n = 170.4 t + 7123	0.9650
318	1.794	ln n = − 1.794 × 10^–2^ t − 7.526	0.9950	386.6	1/n = 386.6 t + 14,729	0.9792
323	1.847	ln n = − 1.847 × 10^–2^ t − 7.175	0.9973	342.2	1/n = 342.2 t + 11,313	0.9857

In the photochemistry system, 1,3-butadiene simultaneously undergoes both thermal and photolysis reactions, under the combined action of the system temperature and ultraviolet light intensity. However, the rates of the thermal and photolysis reactions were different, according to the data in Tables 1 and 2: the thermal activation energy is approximately three times larger than the photolysis one., so the rate of photochemistry should be much greater than thermochemistry in theory [26]. The ultraviolet illumination efficiently promotes the 1,3-butadiene photoreaction. This could indicate that the photolysis would predominate in the photochemistry system, and the thermal reaction could thus be neglected in the kinetic calculations. Therefore, we studied the 1,3-butadiene photolysis under different ultraviolet intensities at 254 nm and various temperatures.

### Effect of light intensity on 1,3-butadiene photoreaction

**Fig. 6:**
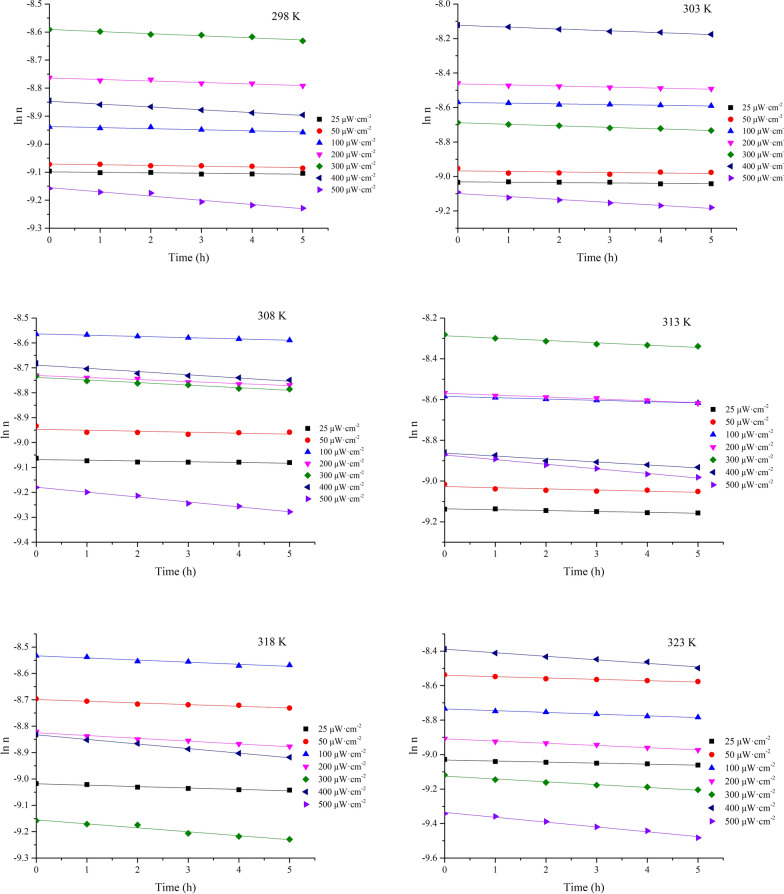
Correlation plots of ln *n* vs. *t* for the photoreaction of 1,3-butadiene at different temperatures

The irradiation intensity is an important factor that influences the photoreaction activity of organic chemicals [27]. Owing to their low cost and accessibility, 254 nm UV lamp plays an important role in daily life and are widely used in disinfection [28] and degradation of organic species [29]. The maximum ultraviolet intensity of outdoor solar radiation at 254 nm was detected as approximately 30 μW cm^−2^. Therefore, the effect of the light intensity on the 1,3-butadiene photoreaction was studied in the temperature and light intensity ranges of 298–323 K and 25–500 μW cm^−2^, respectively. The effect of temperature and ultraviolet intensity on the rate constant of the photochemical reactions is illustrated in Fig. 6.

**Fig. 7:**
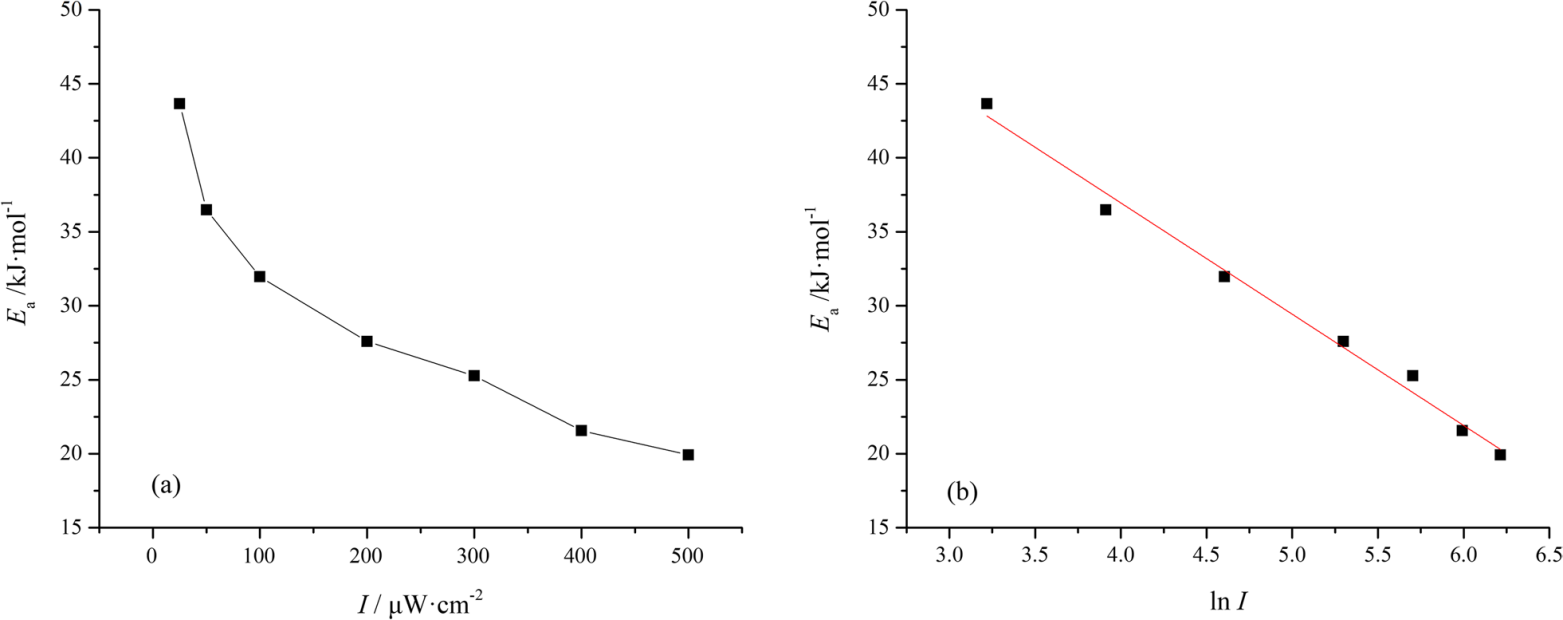
Relationship curve for the activation energy with ultraviolet intensity. **a** *E*_a_ vs. *I*; **b** *E*_a_ vs. ln *I*

The rate constant increased from 0.635 × 10^−2^ to 1.501 × 10^−2^ h^−1^ when the ultraviolet intensity was changed from 25 to 500 μW cm^−2^ at 298 K (Table 3). The increase of the 1,3-butadiene rate constant with the ultraviolet intensity is related to the fact that a higher intensity enables the reactant molecules to gain sufficient energy to cross the energy barrier. As a result, an enhanced reaction rate was obtained. The activation energies for the 1,3-butadiene photoreaction calculated from the Arrhenius Eq. (5) were 19.92–43.65 kJ mol^−1^, and decreased with increasing ultraviolet irradiation (Fig. 7a). A linear relationship was observed between the activation energy and the logarithm of the light intensity (Fig. 7b); the corresponding equation could be expressed as *E*_a_ =  − 7.294·ln*I* + 66.15 (R^2^ = 0.9886). This shows that the ultraviolet light intensity can be controlled to make the reaction proceed in the preferred direction [30, 31].

**Table 3:** Kinetic parameters for the photoreaction of 1,3-butadiene

*I*/(*μ*W cm^−2^)	*k*_2_ × 10^–2^/h^−1^						*E*_a_/(kJ mol^−1^)	R^2^
	298 K	303 K	308 K	313 K	318 K	323 K		
25	0.1635	0.2187	0.2973	0.4333	0.5333	0.5962	43.65	0.9821
50	0.2605	0.3112	0.3958	0.5642	0.6391	0.7764	36.49	0.9865
100	0.3879	0.4088	0.5113	0.6420	0.8020	1.004	31.96	0.9744
200	0.5395	0.6232	0.8182	0.9311	1.064	1.273	27.59	0.9901
300	0.7516	0.9006	1.038	1.146	1.501	1.638	25.26	0.9836
400	1.009	1.077	1.302	1.450	1.739	2.056	21.57	0.9863
500	1.501	1.721	1.974	2.260	2.477	2.804	19.92	0.9815

### Quantum yield of 1,3-butadiene

The quantum yield (*Φ*) is the ratio of the amount of reactant to the number of Einstein absorbed in a certain time, which reflects the efficiency of a photochemical reaction [32, 33]. The number of Einstein absorbed is defined as:

7 $$R\left( {\text{t}} \right) = \frac{{\left( {I_{0} - I_{1} } \right){\text{S}}}}{{N_{A} {\text{hV}}}}{ }$$

where *S* is the irradiated area of 1,3-butadiene (3.5 cm^2^), *I*_0_ and *I*_1_ denote the incident and transmitted light intensity (μW cm^−2^), respectively, *N*_A_ is the Avogadro’s number (6.023 × 10^23^), *V* is the volume of 1,3-butadiene (3.5 cm^3^), *h* is the Planck’s constant (6.63 × 10^−34^ J s), and *ν* is the frequency of the UV light (*ν* = *c*/*λ*, with *c* = velocity of light and *λ* = 254 nm). The amount of reactant was calculated according to the chemical reaction kinetics, giving

8 $${\Phi } = \frac{{ - dn_{BD} /dt}}{R\left( t \right)} = \frac{{ - k_{2} n_{BD} }}{R\left( t \right)}$$

Quantum yield measurements were carried out at 303 K in nitrogen atmosphere. The initial quantum yield of 1,3-butadiene is shown in Table 4; the data show that *Φ* did not vary with the UV light intensity after the initial reaction stage, and its average value was 31.46. It could be inferred that a radical produced by an activated 1,3-butadiene molecule may cause several molecules to react, rather than only one molecule [34].

**Table 4:** The effect of varying ultraviolet intensity on the quantum yield (*Φ*) of 1,3-butadiene

*I*/(*μ*W cm^−2^)	*k*_*2*_/h^−1^	*I*_0_/(*μ*W cm^−2^)	*I*_1_/(*μ*W cm^−2^)	*I*_*0*_–*I*_1_/(*μ*W cm^−2^)	*Φ*
25	0.001670	20	18.7	1.3	37.19
50	0.002417	38	35.2	2.8	24.99
100	0.003418	72	69	3	32.99
200	0.005297	133	127	6	25.56
300	0.007364	207	198	9	23.69
400	0.009555	280	272	8	34.58
500	0.01282	351	342	9	41.24

### Reaction products and pathways of 1,3-butadiene

Another basic task in chemical kinetics is the study of the reaction process. This analysis can reveal the relationship between the structure of a compound and its ability to react, thereby providing a deeper understanding of its chemical transformations. The comparison of the observed mass fragments with the GC–MS library revealed the main products of the dimerization and photolysis reaction of 1,3-butadiene (Figure S1). The relative content of the product was calculated by the peak area normalization method.

**Fig. 8:**
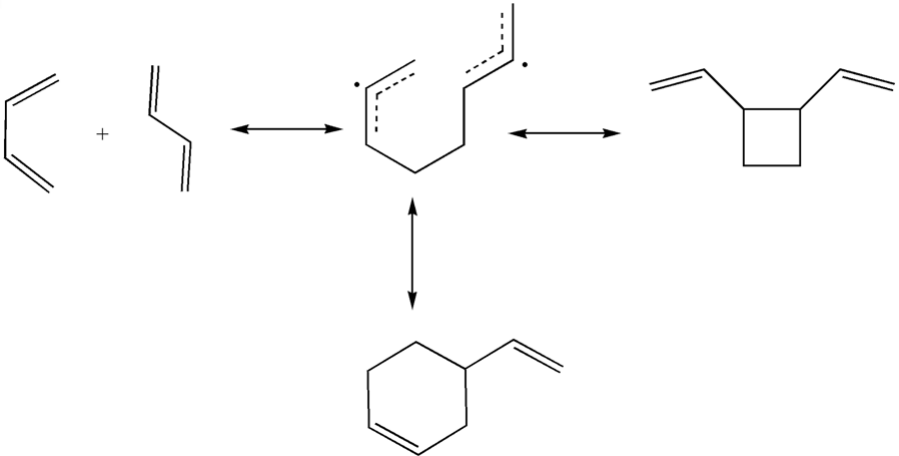
Thermal reaction pathways of 1,3-butadiene

As shown in Table 5, 1,2-divinylcyclobutane and 4-vinylcyclohexene were the main products of the thermal reaction of 1,3-butadiene under nitrogen atmosphere. This result supports the findings of a previous work [15]. 1,3-Butadiene was mainly dimerised at the initial stage of the reaction, and the dimerization proceeded via the Diels–Alder reaction. The proposed reaction pathways shown in Fig. 8 are based on the present experimental results and previous studies [14, 15]. It should be noted that a rotational barrier of 20.10 kJ mol^−1^ separates the *trans* and *cis* isomers of 1,3-butadiene, which enables their rapid conversion [35]. In the dimerization pathway, *trans*- and *cis*-butadiene will dimerise into 1,2-divinylcyclobutane and 4-vinylcyclohexene. These reactions proceed through the formation of the octa-1,7-diene-3,6-diyl radical intermediate [15]. The concerted mechanism suggests that the 1,3-butadiene monomers would directly dimerise into the final product through an activated transition state [36].

**Table 5:** Main identified products of 1,3-butadiene in the thermal reaction

No.	Name	Molecular formula	Relative amount/%	Similarity/%
1	1,3-butadiene	CH_2_ = CH–CH = CH_2_	79.84	98
2	1,2-divinylcyclobutane		0.66	94
3	4-vinycyclohexene		19.50	97

In contrast, 1,3-butadiene yielded widely different products under ultraviolet irradiation at 254 nm, including ethylene, acetylene, cyclobutene, 1-butyne, and 1,2-butadiene, as shown in Table 6. The yields of the various volatile products increased with the irradiation time, in agreement with the kinetics analysis of the photolysis reaction (Figure S2).

**Table 6:** Main identified products of 1,3-butadiene in the photolysis reaction

No.	Name	Molecular formula	Relative amount/%					Similarity/%
			Irradiation time (h)					
			1	2	3	4	5	
1	Ethylene	H_2_C = CH_2_	1.72	1.82	2.23	3.18	4.35	99
2	Acetylene	HC≡CH		1.07	1.23	1.80	2.45	99
3	Cyclobutene		2.63	3.69	4.50	6.56	7.91	92
4	1-Butyne	CH_3_–CH_2_–HC≡CH		0.37	0.45	0.68	0.78	89
5	1,2-Butadiene	CH_3_–CH = C = CH_2_	7.33	7.86	10.28	17.44	18.11	98
6	1,3-Butadiene	CH_2_ = CH–CH = CH_2_	88.31	85.19	81.31	70.34	66.40	98

**Fig. 9:**
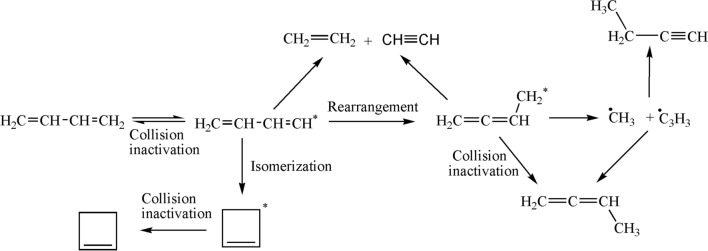
Photolysis reaction pathways of 1,3-butadiene

The photolysis reaction pathways are illustrated in Fig. 9. As discussed above, the variety of products that are formed in the direct photolysis originate from the subsequent photochemical processes. The absorption of ultraviolet irradiation by 1,3-butadiene would lead to the formation of an excited 1,3-butadiene molecule, which may be followed by collisional deactivation to the ground-state molecule, or rearrangement to an excited 1,2-butadiene molecule. The latter may either decompose or be deactivated to a stable 1,2-butadiene molecule through a collision. The main sources for acetylene and ethylene appear to be the excited 1,3-butadiene and 1,2-butadiene molecules. Furthermore, the excited 1,2-butadiene molecule would decompose into CH_3_• and C_3_H_3_• radicals [37]. The secondary reactions of CH_3_• radicals may include recombination to yield ethane [38, 39]. The reaction of the C_3_H_3_• radical is slightly more complicated, due to its uncertain structure. In particular, this radical can adopt two types of structures, CH_2_ = C = CH• and •CH_2_–C≡CH, whose reaction with CH_3_• radicals would yield 1,2-butadiene and 1-butyne, respectively. Although both products have been detected in our measurements, only the presence of the •CH_2_–C≡CH structure could be confirmed, while that of the CH_2_ = C = CH• structure was uncertain, because the source of 1,2-butadiene could be the collision-induced deactivation of the excited 1,2-butadiene molecules [38]. In addition, the photolysis of 1,3-butadiene in the gas phase also leads to the formation of an isomeric product, i.e., cyclobutene. The excited 1,3-butadiene molecule are scrambled by structural isomerization reactions leading to an excited cyclobutene molecule. And then it would be deactivated to a stable cyclobutene molecule through a collision [40].

### Photolysis and photodimerization reactions of 1,3-butadiene

We also observed that the photolysis of 1,3-butadiene at 254 nm in the absence of any photosensitizer was markedly different from the 1,3-butadiene dimerization at 254 nm and from the dimerization initiated by a triplet sensitizer. A possible explanation for this difference might be that the ultraviolet light acted directly on 1,3-butadiene in the gas phase, causing it to undergo a decomposition reaction [20, 41]. According to the first law of photochemistry, only light absorption by molecules can effectively lead to photochemical changes [42]. A photochemical reaction can only proceed when the energy required for the molecule to jump from the ground to the excited state matches the energy of the photon [43]. 1,3-Butadiene has four π molecular orbitals: the lowest two are full, while the other two, with higher energies, are empty. The lowest-energy electron transition of 1,3-butadiene occurs from the *π*_2_ to *π*_3_* orbital at 220 nm, which means that a minimum energy of 544 kJ mol^−1^ is required for the electron transition [44]. Compared with this energy, the transition energy corresponding to the 254 nm irradiation is not high enough, but the energy difference between the two wavelengths is only 73 kJ mol^−1^. Thus, when the irradiation time is long enough, the absorbed light can also break the chemical bonds in the 1,3-butadiene backbone to trigger the photolysis reaction. The generation of photolysis products also proves that gaseous 1,3-butadiene is expected to absorb light at 254 nm, and the reduction in light intensity is not caused by surface reflections of the container. In addition, the photochemistry of 1,3-butadiene in solution, which corresponds to the reaction at very high pressures, yielded none of the main volatile products. An increase in the gas pressure led to a decreased yield of all volatile products; these yields would drop to zero at sufficiently high pressures [20].

Sensitizers have been employed to provide an excited triplet state for 1,3-butadiene to form ring compounds instead of cracking [17]. The presence of the sensitizers makes the cycloaddition reaction easier than the photolysis. At an optimum concentration, photodimerization accounted for less than 10% of the butadiene consumption, and the yield of dimers in the triplet-sensitised reaction was nearly 75%. The main photodimerization products were 1,2-divinylcyclobutane, 4-vinylcyclohexene, and 2-vinylbicyclo[3.1.0]hexane. Approximately 8% of the products consisted of 1,5-cyclooctadiene, which has been reported as a product of the triplet-sensitised reaction [45].

Accurate ab initio calculations of potential energy surfaces for the dissociation and dimerization pathways of 1,3-butadiene are highly complementary to experimental studies of the photodissociation dynamics, because they provide insight into possible reaction products and their energies, as well as into various reaction mechanisms leading to these products. Lee et al. used high-level ab initio calculations to investigate the reaction mechanism of the photodissociation of 1,3-butadiene [46]. The photodissociation of 1,3-butadiene to acetylene and ethylene involved 1,3-hydrogen migration from the terminal CH_2_ group via a transition state, followed by C–C bond cleavage. The energy of the transition state calculated at the G2M level of theory was 366.1 kJ mol^−1^. The C_2_H_2_ + C_2_H_4_ products reside  ~ 167.36 kJ mol^−1^ above 1,3-butadiene. Li et al. carried out ab initio CASSCF calculations to locate transition structures for the dimerization of 1,3-butadiene [47]. 1,3-Butadiene was found to undergo a Diels–Alder reaction to form 4-vinylcyclohexene via a transition structure, with a measured activation energy of 422.17 kJ mol^–1^. The comparison of the activation energies indicated that photodecomposition is more likely to occur than photodimerization.

## Conclusions

We investigated the reactions and kinetics of gaseous 1,3-butadiene under 254 nm ultraviolet irradiation in nitrogen atmosphere at the temperature range from 298 to 323 K. We demonstrated a conceptually different and practical approach for the quantitative analysis of gaseous 1,3-butadiene by UV–vis spectrophotometry in a closed gaseous mini–reactor. Kinetic experiments were performed both without and with UV irradiation of 1,3-butadiene, yielding activation energies of 50.48 and 19.92–43.65 kJ mol^−1^, respectively, which indicated that UV irradiation could accelerate the reaction rate of 1,3-butadiene. Moreover, the possible reaction pathways for the photolysis process were discussed in combination with the identified products. The differences between the dimerization and dissociation processes of 1,3-butadiene under ultraviolet irradiation were elucidated by combining experimental and theoretical methods. In summary, this study provides a feasible approach for the analysis of gaseous products by UV–vis spectrophotometry, paving the way for a more complete understanding of the photochemical reactions of 1,3-butadiene.

## Supplementary Information

**Additional file 1: Figure S1.** Total ion flow diagram of 1,3-butadiene thermal reaction products measured by GC-MS. **Figure S2.** Total ion flow diagram of 1,3-butadiene photolysis reaction products measured by GC-MS.

## Data Availability

All data generated or analysed during this study are included in this published article (and its Additional files).
