# Supplementary material for: Reactivity and kinetics of 1,3-butadiene under ultraviolet irradiation at 254 nm
Source: BMC Chem. 2022 Feb 18;16(1):4. doi: 10.1186/s13065-022-00800-6 (PMC8857861; doi:10.1186/s13065-022-00800-6)
Supplement: Supplementary file 1 — Additional file 1: Figure S1. Total ion flow diagram of 1,3-butadiene thermal reaction products measured by GC-MS. Figure S2. Total ion flow diagram of 1,3-butadiene photolysis reaction products measured by GC-MS. [file 13065_2022_800_MOESM1_ESM.pdf]

Supplementary Material

Reactivity and kinetics of 1,3-butadiene under ultraviolet irradiation at 254 nm

Min Liang, Chang Yu, Suyi Dai, Haijun Cheng, Weiguang Li, Fang Lai, Li Ma*, Xiongmin Liu*

School of Chemistry and Chemical Engineering, Guangxi University, Nanning 530004, China

*Corresponding author. E–mail address: gxumali@126.com (L.M.); xmliu1@gxu.edu.cn (X.L.)

This PDF file includes:

Figs.S1-S2

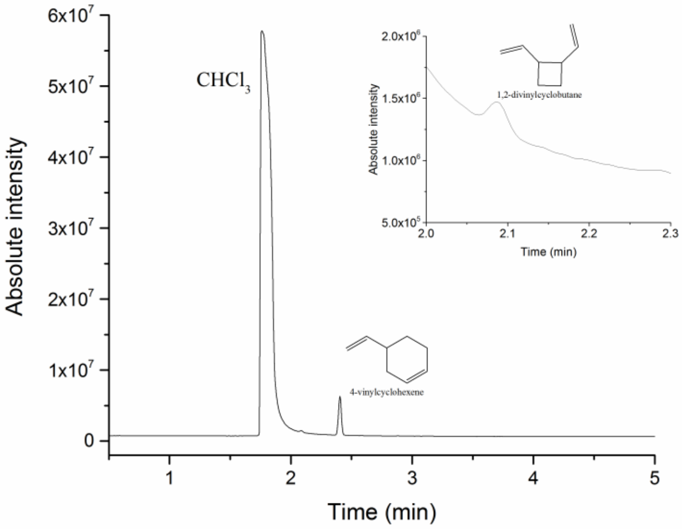

Fig.S1 Total ion flow diagram of 1,3-butadiene thermal reaction products measured by GC-MS.

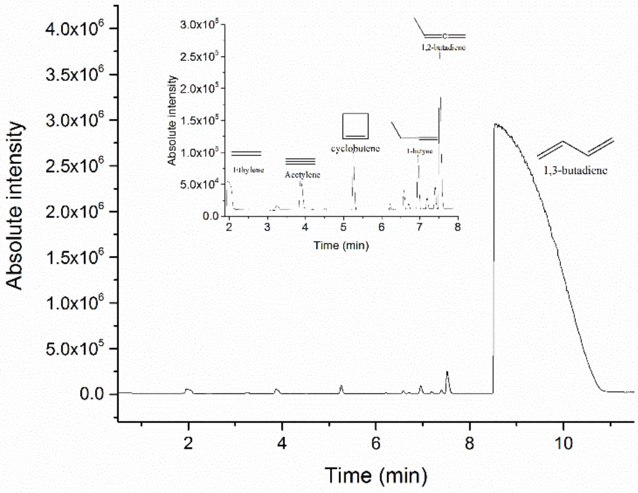

Fig.S2 Total ion flow diagram of 1,3-butadiene photolysis reaction products measured by GC-MS.
